# Survival Benefit of Anticoagulation Therapy in End Stage Kidney Disease Patients with Atrial Fibrillation: A Single Center Retrospective Study

**DOI:** 10.3390/medicina58010058

**Published:** 2021-12-30

**Authors:** Mi-Ryung Kim, Deok-Gie Kim, Han-Wul Shin, Sung-Hwa Kim, Jae-Seok Kim, Jae-Won Yang, Byoung-Geun Han, Seong-Ok Choi, Jun Young Lee

**Affiliations:** 1 Department of Nephrology, Yonsei University Wonju College of Medicine, Wonju 26426, Korea; miryung2@yonsei.ac.kr (M.-R.K.); dragonshw@yonsei.ac.kr (H.-W.S.); ripplesong@yonsei.ac.kr (J.-S.K.); kidney74@yonsei.ac.kr (J.-W.Y.); neptune@yonsei.ac.kr (B.-G.H.); kidney77@yonsei.ac.kr (S.-O.C.); 2 Department of Surgery, Yonsei University Wonju College of Medicine, Wonju 26426, Korea; exdelcomp20@gmail.com; 3 Department of Biostatistics, Yonsei University Wonju College of Medicine, Wonju 26426, Korea; juniver1057@naver.com; 4 Center of Evidence Based Medicine, Institute of Convergence Science, Yonsei University, Seoul 03722, Korea

**Keywords:** anticoagulation, atrial fibrillation, end-stage kidney disease

## Abstract

*Background and Objectives*: Although the need for anticoagulation to prevent thromboembolism is increasing and non-vitamin K antagonist oral anticoagulants (NOACs) have been tried, there is still controversy about the efficacy of anticoagulation in patients with dialysis. *Materials and Methods*: We retrospectively analyzed the risk and benefit of anticoagulation in dialysis patients with atrial fibrillation (AF). We retrospectively analyzed all data of 89 patients who received dialysis therapy and were diagnosed with AF. Among them, 27 received anticoagulation (11 warfarin and 16 apixaban 2.5 mg twice a day), while 62 received no anticoagulation. *Results*: In multivariate Cox regression analysis, compared to no anticoagulation treatment, anticoagulation treatment was associated with a low incidence of all-cause mortality (hazard ratios (HR) 0.36; 95% confidence interval (CI) 0.15–0.88). Compared to no anticoagulation treatment, more anticoagulation treatment patients experienced severe bleeding (HR 4.67; 95% CI 1.26–17.25) and any bleeding (HR 2.79; 95% CI 1.01–7.74). Compared to no anticoagulation, warfarin treatment patients were associated with a low incidence of all-cause mortality (HR 0.26; 95% CI 0.09–0.81) and a high incidence of severe bleeding (HR 4.85; 95% CI 1.12–21.10). All-cause mortality and bleeding were not significantly different between no anticoagulation and apixaban treatment patients. *Conclusions*: In dialysis patients with AF, anticoagulation therapy is associated with an increased incidence of severe bleeding, but anticoagulation therapy is associated with a low incidence of all-cause mortality. Individualized anticoagulation therapy with careful bleeding monitoring is needed in dialysis patients with AF.

## 1. Introduction

Atrial fibrillation (AF) belongs to the category of supraventricular arrhythmia, and over 7–13% of patients on dialysis have this disease, which is 10–20-fold higher than the general population [1,2]. Patients with AF have a 4–5 times higher risk of stroke, thromboembolism, and mortality risk for patients on dialysis [2,3]. Although the need for anticoagulation in patients with atrial AF on dialysis is increasing, there has been no randomized controlled trial (RCT) regarding the use of anticoagulation in this population. Moreover, the use of anticoagulation in patients on dialysis is still controversial [4,5].

Because of the effectiveness and safety of non-vitamin K antagonist oral anticoagulants (NOACs) and the fact that these do not require routine monitoring of coagulation, these are emerging as a replacement for warfarin in stroke and thromboembolism treatment [6,7,8,9,10]. Four types of NOACs are currently used: dabigatran, rivaroxaban, apixaban, and edoxaban [7,8,9,10]. However, the efficacy of NOACs has not been fully proven, and several severe side effects, such as intracranial bleeding and gastrointestinal bleeding, were reported in patients on dialysis [11,12].

Among the four types of NOACs, only apixaban received approval from the Food and Drug Administration (FDA); the number of patients with AF on dialysis receiving apixaban increased [13]. However, the FDA approved apixaban based on pharmacokinetic data, not clinical outcomes [14]. Following this trend, the AHA/ACC/HRS guideline in 2019 described the use of apixaban as a feasible option in patients with AF and on dialysis [15]. Nevertheless, the efficacy, proper dose, and risk of apixaban in patients with AF on dialysis are not yet clearly documented. Thus, we conducted a retrospective study to clarify the effectiveness and risk of anticoagulation therapy in patients with AF on dialysis.

## 2. Materials and Methods

### 2.1. Study Design and Subject

We conducted a retrospective cohort study of patients who were diagnosed with AF or atrial flutter and on dialysis in Wonju Severance Christian Hospital. Data were collected from the electronic medical record system of Wonju Severance Christian Hospital from 2010 to 2020. This study was approved by the Institutional Review Board of Wonju Severance Christian Hospital (CR320114). Written consent from subjects was not necessary for this is a retrospective study. We included 182 patients who had a diagnostic code and AF or atrial flutter on electrocardiography and simultaneously had hemodialysis or peritoneal dialysis during the study period. We excluded subjects with a history of cancer (n = 37), those who received kidney transplantation (*n* = 4), those who had a short-term follow-up period (within 90 days) (*n* = 21), who received mechanical valve replacement therapy (*n* = 11), and those who had missing laboratory values (*n* = 21). Finally, 89 patients (16 peritoneal dialysis patients and 73 hemodialysis patients) were enrolled in this study. According to whether they were taking anticoagulant medication (apixaban 2.5 mg twice a day or warfarin) or not, patients were classified into two groups (anticoagulation group and no anticoagulation group). Patients receiving anticoagulant therapy were divided into two groups according to type or drug (apixaban or warfarin). Among the 27 patients in the anticoagulation group, 16 had been prescribed apixaban 2.5 mg twice a day, and 11 had been prescribed warfarin.

### 2.2. Data Collection

Demographic variables, including age, gender, and various medical histories, for example, history of major bleeding (gastrointestinal and cerebral), thromboembolism, major adverse cardiovascular events (MACE), and hospitalization, were collected from the database. Baseline laboratory profiles were measured at the index date. The CHA_2_DS_2_-VASc (consisting of congestive heart failure, hypertension, age, diabetes, previous stroke/transient ischemic attack, vascular disease, and sex) and HAS-BLED (consisting of hypertension, abnormal renal and liver function, stroke, bleeding history, the labile international normalized ratio (INR), elderly, and drugs or alcohol) scores and Charlson comorbidity index (CCI) were calculated at the same time [16]. Previous medication history was reviewed by researching medical records. Platelet aggregation inhibitors, warfarin, and other all kinds of bleeding tendency-related medications were reviewed. All kinds of other prescribed medications, including antihypertensive medications, were also reviewed and included. We defined the index date only when the patients were on dialysis and diagnosed with AF or atrial flutter. We defined the primary outcome as all-cause mortality and cardiovascular mortality. The secondary outcome included MACE, and adverse events include severe bleeding events (gastrointestinal bleeding with admission or cerebrovascular bleeding or other life-threatening bleeding), stroke, any thromboembolism, deep vein thrombosis, and transient ischemic attack.

### 2.3. Statistical Analysis

Continuous variables are presented as means and standard deviations, and categorical variables are presented as frequencies and percentages. The two-sample *t*-test, x^2^ test (Fisher exact test), and Mann–Whitney test were used to compare groups as appropriate. Multivariate Cox regression was performed using age, sex, and the CCI, CHA_2_DS_2_-VASc, HAS-BLED scores, and dialysis duration. These variables were chosen considering collinearity and clinical importance. Hazard ratios (HR), 95% confidence intervals (CI), and *p*-values were also shown. Because there was no cardiovascular mortality in the apixaban treatment group, it was impossible to calculate the HR about cardiovascular mortality. The goodness of fit of the model was assessed using the Hosmer–Lemeshow test. A *p*-value of <0.05 was considered statistically significant. All statistical analyses were conducted using IBM Statistical Package for the Social Sciences version 25.0 (IBM Corporation, Armonk, NY, USA).

## 3. Results

### 3.1. Baseline Characteristics

Among the 89 patients, 27 were taking an anticoagulation medication (apixaban 2.5 mg twice a day or warfarin), whereas the other 62 were not. There were no patients who were taking apixaban 5 mg twice a day or other kinds of NOACs. The mean follow-up duration was 879.2 ± 873.5 days. The baseline characteristics were similar between the two groups. Age, dialysis durations, INR prolongation, frequencies of blood transfusion more than three times, INR level, low-density lipoprotein level, CHADVAS score, HAS-BLED score, and prescribed vintage of aspirin and proton pump inhibitors were significantly different between the two groups (Table 1).

### 3.2. Comparison of Clinical Outcomes between Anticoagulation and No Anticoagulation Dialysis Patients

During the follow-up period, the incidences of all-cause mortality and cardiovascular mortality were not statistically different between the anticoagulation and no anticoagulation patients. The incidence of MACE was significantly lower in anticoagulation patients than in no anticoagulation patients (25.9% vs. 43.5%, *p* = 0.009). The incidence of admission, hospitalization, and bleeding-related outcomes was not significantly different between the anticoagulation and no anticoagulation patients (Table 1).

**Figure 1:**
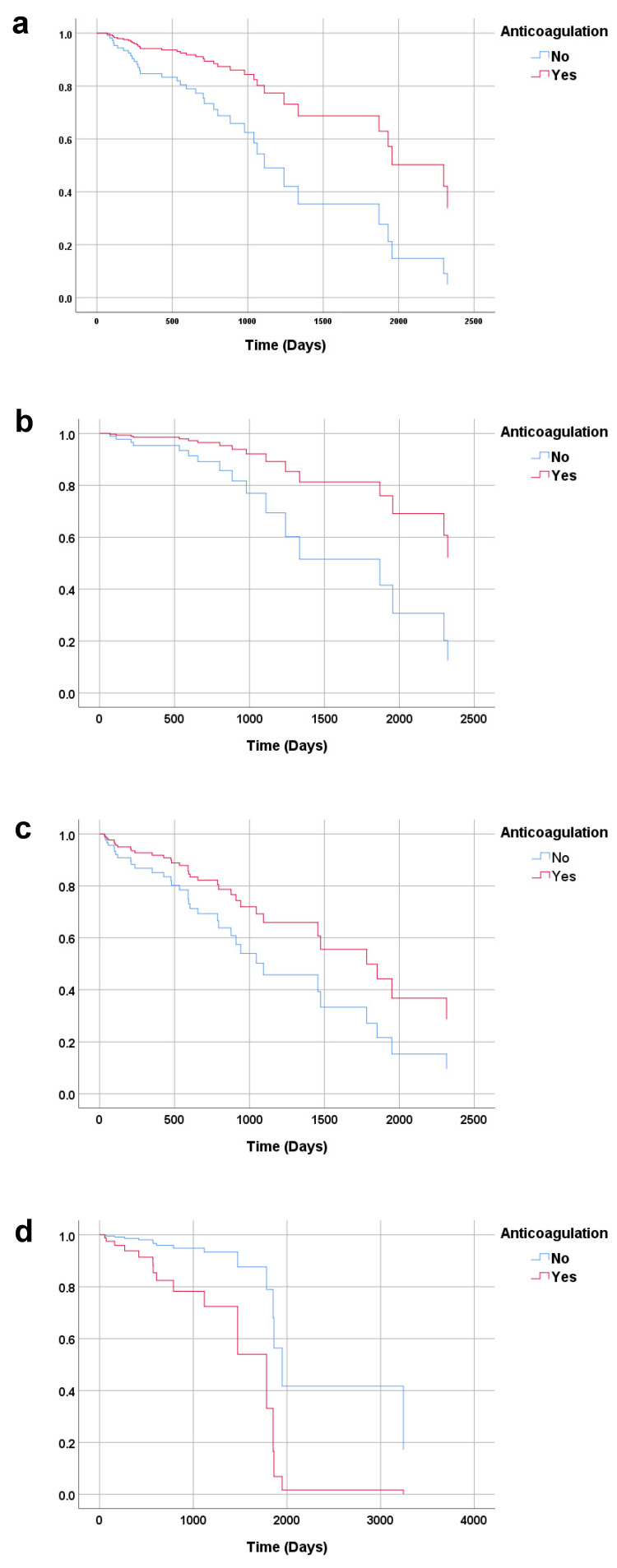

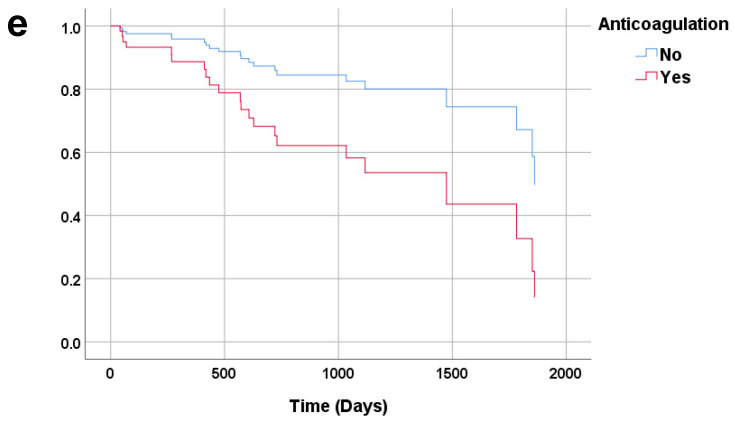
Kaplan–Meier curve analyses for the cumulative incidence of each outcome. (**a**) All-cause mortality (*p* = 0.026). (**b**) Cardiovascular mortality (*p* = 0.313). (**c**) Major cardiovascular adverse events (*p* = 0.166). (**d**) Severe bleeding (*p* = 0.021). (**e**) Any bleeding (*p* = 0.047).

The multivariate-adjusted Cox regression analysis and the Kaplan–Meier curve analysis showed that, compared with no anticoagulation treatment, anticoagulation treatment was associated with a low incidence of all-cause mortality (HR 0.36; 95% CI 0.15–0.88) and a high incidence of severe bleeding (HR 4.67; 95% CI 1.26–17.26) and any bleeding (HR 2.81; 95% CI, 1.01–7.81) (Figure 1).

Cardiovascular mortality, MACE, brain hemorrhage, brain infarction, and gastrointestinal bleeding were not significantly different between the anticoagulation patients and no anticoagulation patients (Table 2) (Figure 1).

### 3.3. Comparison of Clinical Outcomes between Anticoagulation and No Anticoagulation Dialysis Patients, Subgroup Analysis

The multivariate-adjusted Cox regression analysis also showed that, compared with no anticoagulation, warfarin treatment was associated with a reduced incidence of all-cause mortality (HR 0.26; 95% CI 0.09–0.8) and an increased incidence of severe bleeding (HR 4.86; 95% CI 1.21–21.12). Cardiovascular mortality, MACE, any bleeding, brain infarction, brain hemorrhage, and gastrointestinal bleeding were not significantly different between warfarin treatment and no anticoagulation patients (Table 3).

All-cause mortality, MACE, severe bleeding, any bleeding, brain hemorrhage, brain infarction, and gastrointestinal bleeding were not significantly different between no anticoagulation and apixaban treatment patients in the multivariate-adjusted Cox regression analysis (Table 4).

## 4. Discussion

In our study, compared with the no anticoagulation group, the anticoagulation group was associated with reduced incidence of all-cause mortality and increased incidence of severe bleeding and any bleeding. In the subgroup analysis, warfarin treatment, compared with no anticoagulation, also reduced the incidence of all-cause mortality and increased incidence of any bleeding.

Unfortunately, there are no (RCT)s for evaluating the long-term efficacy and safety of anticoagulation in dialysis patients with AF and there are also no RCTs for the use of alternative stroke prevention strategies, such as left arterial appendage occlude implantation for these individuals. Several observational cohort studies and meta-analyses showed conflicting results for the use of anticoagulation therapy (warfarin and NOACs) in dialysis patients with AF [12,13,17,18,19,20,21,22,23,24,25,26]. Among those studies, only one study showed that compared with no anticoagulation therapy, warfarin therapy increased all-cause mortality dialysis patients with AF [25]. However, those studies included 26.4% of CHA_2_DS_2_-VASc scores below 2 points in patients who did not need anticoagulation therapy according to recent guidelines [5,27]. Two recently published studies reported that warfarin and apixaban (5 mg twice a day) reduced all-cause mortality in dialysis patients with AF [20,26]. However, most other studies did not show a difference of all-cause mortality between anticoagulation treatment and no anticoagulation dialysis patients with AF [18,21,23,24]. Reducing the incidence of stroke, selection bias of anticoagulation patients, warfarin-associated vascular calcification, and time in the therapeutic range of warfarin treatment patients might be associated with this phenomenon, but the exact mechanism needs more research [26]. Reflecting recent guidelines, our study applied the modified CHA_2_DS_2_-VASc score, inclusion criteria, and sexual difference of the cut-off value of the start of anticoagulation and showed a mortality benefit [27].

Anticoagulation therapy may increase the risk of major bleeding in dialysis patients with AF [17,20,21,23,24]. In the general AF population, the HAS-BLED score had the best evidence for predicting bleeding risk [27]. However, in dialysis patients with AF, several bleeding risks scores, including the HAS-BLED score, showed poor predictive abilities [28]. As shown in our study, anticoagulation therapy may increase bleeding, but it did not seem to bring about an increase in all-cause mortality. Therefore, several recently published guidelines and review articles recommend dialysis patients with AF need individualized anticoagulation therapy considering bleeding risk and stroke risk [5,15,27,29,30].

Warfarin is the most widely used and only one option of anticoagulation agents in dialysis patients with AF in the past. As patients on dialysis have a higher bleeding tendency, warfarin may increase bleeding events; major bleeding complications are the main concern in administering warfarin in those patients [17]. It is difficult to control the INR level within the therapeutic range because uremia interferes with warfarin metabolism through hepatic p450 [31]. Compared to warfarin, 5 mg of apixaban showed a mortality benefit in recently published data [13]. Accordingly, there is a significant decrease in the number of warfarin used per year, with an increase of NOACs use, predominantly apixaban [13]. Nevertheless, some dialysis patients with AF still need to use warfarin for economic or medical (mechanical valve) reasons.

This retrospective observational cohort study has several limitations. This study was a single center retrospective study with a relatively small number of patients. As the number of patients prescribed with apixaban was small, our study had limited explanatory power. In addition, compared with warfarin, apixaban is a new drug that was prescribed in our hospital only after 2017, so apixaban-treated patients had a relatively short follow-up duration compared to the warfarin-treated patients. Despite these limitations, the strength of our study is that we reviewed as many medications as possible that could affect the bleeding potency, including antiplatelet and antihypertensive agents and diabetes medications. In addition, our study calculated CHA_2_DS_2_-VASc scores according to the latest guidelines. These points could reduce other major biases that could have affected cardiovascular outcomes.

For dialysis patients with AF, anticoagulation increases the risk of bleeding, regardless of the type of medication. Our study showed that compared with no anticoagulation, anticoagulation reduced the incidences of all-cause mortality, but the incidences of major bleeding also increased. Through our results, we could recommend individualized anticoagulation therapy in patients with ESKD, with careful bleeding monitoring, and further large studies are needed.

## Figures and Tables

**Table 1:** Baseline characteristics of anticoagulation and no anticoagulation patients.

	Anticoagulation (*n* = 27)	No Anticoagulation (*n* = 62)	*p*-Value
Age (years)	66.4 ± 11.7	72.1 ± 11.9	0.039
Sex (male, %)	13 (48.1)	35 (56.5)	0.496
HD (*n*, %)	23 (85.2)	50 (80.6)	0.768
BMI	23.6 ± 3.4	22.8 ± 4.0	0.374
Dialysis duration (days)	1737.1 ± 1662.5	2980.0 ± 2428.3	0.018
HTN (*n*, %)	27 (100)	61 (98.4)	0.697
DM (*n*, %)	10 (37.0)	34 (54.8)	0.167
Bleeding Hx (*n*, %)	4 (14.8)	5 (8.1)	0.446
HF (*n*, %)	11 (40.7)	22 (35.5)	0.604
Old CVA (*n*, %)	7 (25.9)	8 (12.9)	0.116
LC (*n*, %)	3 (11.1)	6 (9.7)	0.553
INR > 3 (*n*, %)	11 (40.7)	0 (0)	<0.001
Transfusion ≥ 3 units	6 (22.2)	1 (1.6)	0.003
EF (%)	54.6 ± 14.2	56.9 ± 11.9	0.449
BNP (pg/mL)	1904.2 ± 1408.0	1852.5 ± 1600.4	0.892
Hb (g/dL)	10.4 ± 1.5	10.3 ± 1.4	0.824
Platelet count (E9/L)	204.5 ± 84.0	191.0 ± 59.9	0.391
AST (U/L)	34.4 ± 46.4	21.4 ± 9.4	0.036
ALT (U/L)	37.9 ± 90.5	17.0 ± 11.8	0.076
Total bilirubin (mg/dL)	0.6 ± 0.7	0.9 ± 2.7	0.645
INR	1.6 ± 0.7	1.0 ± 0.1	<0.001
LDL (mg/dL)	60.2 ± 27.0	81.3 ± 43.7	0.008
CRP (mg/dL)	3.5 ± 5.8	3.1 ± 5.5	0.747
Medication			
Aspirin (*n*, %)	7 (25.9)	37 (59.7)	0.003
Clopidogrel (*n*, %)	3 (11.1)	17 (27.4)	0.074
Other medication * (*n*, %)	4 (14.8)	7 (11.3)	0.441
Statin (*n*, %)	10 (38.0)	28 (45.2)	0.317
PPI (*n*, %)	11 (40.7)	10 (16.4)	0.016
NSAID (*n*, %)	0 (0)	3 (4.8)	0.333
HAS-BLED score	4.2 ± 0.7	3.6 ± 0.9	0.001
CHAD-VAS score	4.4 ± 1.2	3.7 ± 1.6	0.025
CCI index	7.0 ± 2.3	6.2 ± 1.9	0.112
Brain infarction (*n*, %)	1 (3.7)	7 (11.3)	0.236
Brain hemorrhage (*n*, %)	3 (11.1)	4 (6.5)	0.358
GI bleeding (*n*, %)	4 (14.8)	6 (9.7)	0.355
Severe bleeding (*n*, %)	8 (29.6)	8 (12.9)	0.059
Any bleeding (*n*, %)	10 (37.0)	12 (19.4)	0.068
Hospitalization (*n*, %)	2.3 ± 3.2	3.0 ± 2.9	0.319
Admission (*n*, %)	1.7 ± 2.1	2.2 ± 2.1	0.321
All-cause mortality (*n*, %)	7 (25.9)	29 (46.8)	0.052
CV mortality (*n*, %)	3 (11.1)	14 (22.6)	0.166
MACE (*n*, %)	7 (25.9)	27 (43.5)	0.009

Abbreviations: ALT, alanine aminotransferase; AST, aspartate aminotransferase; BMI, body mass index; BNP, brain natriuretic peptide; CABG, coronary artery bypass graft; CAOD, coronary artery disease; CCI, Charlson comorbidity index; CRP, C-reactive protein; CV, cardiovascular; CVA, cerebrovascular accident; DM, diabetes mellitus; EF, ejection fraction; F/U, follow-up; Fx, fracture; Hb, hemoglobin; HD, hemodialysis; HF, heart failure; HTN, hypertension; Hx, history; GI, gastrointestinal; INR, international normalized ratio; LC, liver cirrhosis; LDL, low-density lipoprotein; MACE, major adverse cardiovascular events; N, number; NSAID, nonsteroidal anti-inflammatory drugs; PPI, proton pump inhibitor. * Other medication: Other bleeding-related medication.

**Table 2:** Comparison of any anticoagulation and no anticoagulation.

	Crude	Model 1	Model 2
All-cause mortality	0.77 (0.32–1.65)	0.66 (0.19–1.14)	0.36 (0.15–0.88)
Cardiovascular mortality	0.63 (0.18–2.19)	0.56 (0.16–2.01)	0.31 (0.08–1.26)
MACE	0.78 (0.34–1.80)	0.75 (0.33–1.75)	0.53 (0.22–1.30)
Severe bleeding	4.88 (1.65–14.44)	4.63 (1.54–13.91)	4.67 (1.26–17.25)
Any bleeding	2.71 (1.16–6.35)	2.53 (1.06–6.00)	2.79 (1.01–7.74)
GI bleeding	2.20 (0.61–7.93)	2.09 (0.57–7.73)	3.10 (0.61–15.68)
Brain hemorrhage	3.26 (0.70–15.10)	3.25 (0.69–15.41)	1.86 (0.30–11.68)
Brain infarction	0.38 (0.05–3.12)	0.35 (0.04–2.96)	0.25 (0.03–2.43)

Abbreviation: GI, gastrointestinal; MACE, major adverse cardiovascular events. Model 1: adjusted for age sex. Model 2: adjusted for Model 1 + Charlson comorbidity index and CHA_2_DS_2_-VASc and HAS-BLED scores and dialysis duration.

**Table 3:** Comparison of warfarin anticoagulation and no anticoagulation.

	Crude	Model 1	Model 2
All-cause mortality	0.57 (0.20–1.65)	0.53 (0.18–1.55)	0.26 (0.09–0.81)
Cardiovascular mortality	0.79 (0.23–2.80)	0.73 (0.20–2.62)	0.40 (0.10–1.65)
MACE	0.65 (0.23–1.87)	0.67 (0.23–1.95)	0.44 (0.14–1.37)
Severe bleeding	5.19 (1.64–16.47)	4.95 (1.54–15.91)	4.85 (1.12–21.10)
Any bleeding	2.38 (0.88–6.46)	2.18 (0.79–6.04)	2.08 (0.63–6.87)
GI bleeding	2.50 (0.61–10.17)	2.37 (0.57–9.95)	3.24 (0.54–19.38)
Brain hemorrhage	2.66 (0.48–14.85)	2.62 (0.47–14.66)	2.06 (0.19–22.16)

Abbreviation: GI, gastrointestinal; MACE, major adverse cardiovascular events. Model 1: adjusted for age sex. Model 2: adjusted for Model 1 + Charlson comorbidity index and CHA_2_DS_2_-VASc and HAS-BLED scores and dialysis duration.

**Table 4:** Comparison of apixaban anticoagulation and no anticoagulation.

	Crude	Model 1	Model 2
All-cause mortality	1.29 (0.38–4.35)	1.12 (0.33–3.82)	0.71 (0.20–2.57)
MACE	1.15 (0.34–3.88)	1.05 (0.31–3.56)	0.83 (0.24–2.92)
Severe bleeding	3.44 (0.65–18.25)	3.12 (0.58–16.79)	5.35 (0.73–39.35)
Any bleeding	2.30 (0.63–8.35)	1.98 (0.53–7.33)	2.85 (0.60–13.65)
GI bleeding	1.65 (0.19–14.07)	1.32 (0.15–11.59)	10.15 (0.44–234.07)
Brain hemorrhage	4.86 (0.39–60.16)	4.95 (0.38–64.08)	5.20 (0.29–92.12)
Brain infarction	2.16 (0.23–20.08)	1.99 (0.21–19.19)	1.76 (0.17–18.03)

Abbreviation: MACE, major adverse cardiovascular events. Model 1: adjusted for age sex. Model 2: adjusted for Model 1 + Charlson comorbidity index and CHA_2_DS_2_-VASc and HAS-BLED scores and dialysis duration.

## Data Availability

The data presented in this study are available on request from the corresponding author.
